# Caregiver Burden and Its Relationship to Health-Related Quality of Life in Craniopharyngioma Survivors

**DOI:** 10.1210/clinem/dgad488

**Published:** 2023-08-19

**Authors:** Nathalie Kayadjanian, Eugenie A Hsu, Amy M Wood, Dean S Carson

**Affiliations:** Raymond A. Wood Foundation, Ocean City, MD 21842, USA; Raymond A. Wood Foundation, Ocean City, MD 21842, USA; Raymond A. Wood Foundation, Ocean City, MD 21842, USA; Raymond A. Wood Foundation, Ocean City, MD 21842, USA

**Keywords:** craniopharyngioma, survivor, caregiver burden, health-related quality of life, hyperphagia, poly-symptomatology

## Abstract

**Context:**

Craniopharyngioma (CP) is a rare brain tumor associated with severe comorbidities that reduce survivor health-related quality of life (HRQOL). However, CP impact on caregivers is unknown.

**Objective:**

To measure caregiver burden and examine its relationship to survivor HRQOL and other determinants in CP.

**Methods:**

Eighty-two participants who self-identified as caregivers of CP survivors responded to an online survey including caregiver-reported Pediatric Quality of Life Inventory (PedsQL), and the Zarit Burden Interview (ZBI).

**Results:**

Caregivers reported an average of 13 out of 29 health conditions in survivors following tumor treatment, including excess weight, hypopituitarism, fatigue, mood, cognition, social issues, temperature dysregulation, visual impairment, and sleep problems. Strikingly, 70% of survivors who experienced obesity also experienced hyperphagia. ZBI scores were moderate with a median of 37. PedsQL total scores were poor with a median of 46.2. ZBI scores were independent of caregiver level of education and care duration. Both scores were independent of income, survivor age, gender, age at diagnosis, or tumor recurrence. In contrast, both scores depended on the number (*P* < .001) and the type of survivor health problems, with significantly worse scores for caregivers or survivors with symptoms of hypothalamic dysfunction (*P* < .001) including hyperphagia but not obesity. PedsQL total scores significantly predicted ZBI scores (*P* < .001).

**Conclusion:**

Survivor poly-symptomatology predicted and incurred significant caregiver burden. Our study separated hyperphagia and obesity and identified hyperphagia and other hypothalamic dysfunction symptoms as understudied issues. Altogether, these findings draw particular attention to the unmet needs of CP survivors and their caregivers.

Craniopharyngioma (CP) is a rare brain tumor of embryogenic epithelial origin that arises in the sellar and/or parasellar region and develops from remnants of Rathke's pouch (1). In the United States, the incidence is 0.16 per 100 000 persons, with an increased incidence in Black individuals compared to those of other races (2, 3) but no difference based on gender or geographical location (4, 5). Mortality is 3 to 5 times higher than the general population (6). CP occurrence has a bimodal age distribution with peak incidence rates in children 5 to 9 years of age and adults 55 to 69 years of age. Childhood-onset CP frequently manifests as a chronic disease so that patients require lifelong, continuous care by experienced multidisciplinary teams to manage clinical consequences (1, 7). It has been associated with the lowest quality of life scores of any pediatric brain tumor (8).

Despite being classified as a benign tumor by the World Health Organization and having a 5-year survival rate of 92%, CP and its treatment leave survivors with numerous lifelong adverse effects (1). Its infiltrative tendencies and anatomical proximity to the pituitary gland, hypothalamus, optic pathways, the circle of Willis, and the third ventricle, as well as its high recurrence rate (9), pose the risk of significant chronic neurologic, metabolic, and endocrine comorbidities (10). The tumor is typically treated with gross total resection (GTR) or partial resection surgery (PRS), with or without radiotherapy. However, the best practices for CP treatment are controversial due to the higher risk of tumor recurrence with PRS and the increased risk of adverse iatrogenic effects with GTR. Clinical manifestations resulting from the tumor and/or tumor treatment are multiple and often lifelong (10, 11). Comorbidities include panhypopituitarism, hypothalamic dysfunction, psychosocial problems, cognitive impairment, and visual deficits (12, 13). While hormone replacement therapies can address endocrinopathies, many of the health sequelae cannot be corrected due to the lack of effective treatments (14), thereby severely impairing the quality of life (QOL) of survivors (15).

Hypothalamic dysfunction is associated with an abysmal prognosis (16) and is the strongest predictor of lower QOL in CP survivors (7, 17). Hypothalamic dysfunction results in impaired regulation of temperature, thirst, hunger, sleep/wake cycles, social interactions, mood (18, 19), metabolism, and energy (20, 21) and causes hypothalamic obesity (HO) in about half of all CP survivors (22, 23). No effective treatments are available to address HO or other symptoms of hypothalamic dysfunction (24). Additionally, neurocognitive and psychosocial impairments are prevalent in CP survivors (13, 25). These chronic problems are lacking in treatment and are associated with poor QOL (1, 7, 12, 26).

The aftermath of a CP diagnosis and treatment can be challenging not only for survivors but also for their caregivers who must subsume the day-to-day responsibilities of managing survivors’ chronic illnesses at home. These responsibilities include administering medications, attending medical appointments, helping with basic activities of daily living, and managing cognitive, psychosocial, and behavioral issues. Furthermore, some comorbidities associated with CP can be life-threatening, requiring constant monitoring of symptoms to prevent medical destabilization, as is the case with adrenal crisis or hypo/hypernatremia (20). The tasks involved in caring for CP survivors are demanding, complex, and lifelong, and they can significantly and adversely affect caregiver QOL. To our knowledge, there is only one publication to date showing distress experienced by parents of children newly diagnosed with CP following tumor-directed treatment (27). Therefore, it is important to understand the potential negative impacts of caring for CP survivors as the burden can adversely affect the well-being of caregivers and impact the quality of care and outcome for CP survivors (28, 29).

The goal of this study was to assess the impact of CP on caregivers. We chose to measure caregiver burden, a complex and multidimensional concept, to capture the level of multifaceted strain perceived by the caregiver from caring for CP survivors over an extended period (30). We used the Zarit Burden Interview (ZBI) to measure the subjective burden in caregivers of CP survivors. The ZBI is a widely used validated instrument that measures caregiver burden in various disabilities and chronic illnesses. Additionally, we assessed the determinants of caregiver burden in CP by examining factors that could be associated with caregiver burden including caregiver-reported survivor`s clinical characteristics and health-related quality of life (HRQOL). To measure HRQOL, we utilized the Pediatric Quality of Life Inventory™ (PedsQL™), a commonly used tool for assessing HRQOL in individuals with disabilities (31). Finally, we explored the relationship between caregivers’ perception of survivors’ HRQOL and caregiver burden, as well as investigated whether HRQOL can predict caregiver burden.

## Methods

### Participants

Participants were self-identified caregivers of hypothalamic-pituitary brain tumor survivors aged 5 and older. Caregivers were recruited from brain tumor support groups on Facebook and via the Raymond A. Wood Foundation listserv. A caregiver was defined as having intimate knowledge and involvement in the activities of daily living of a hypothalamic-pituitary brain tumor survivor. Out of the 128 responses received, 108 surveys were completed and examined, and 89 responses identified from caregivers of survivors with CP tumors were included in this study. Of those, 7 with incongruent differences between the age of caregivers and the survivors were excluded, resulting in a total of 82 respondent surveys being analyzed.

### Assessment

The online survey was developed using Survey Monkey software and pretested before it was open to all caregivers. Anonymous data were collected between August 2, 2022, and October 3, 2022. The study was approved by the WCG institutional review board committee (#20212846). The survey consisted of 76 questions including 31 questions on demographics of survivors and their caregivers and clinical characteristics of CP survivors, 23 questions of the Pediatric Quality of Life Inventory (PedsQL), and 22 questions of the Zarit Burden Interview (ZBI).

### Clinical Characteristics of Survivors

Caregivers were asked to provide information on medical and health background on the survivors for whom they care for, including age at diagnosis, age at time of first treatment, number of tumor recurrences, tumor treatment(s), symptoms that occurred following tumor treatment(s), current medications, and medical procedures or interventions the survivor had experienced since diagnosis. To assess the post-CP sequelae symptoms, we generated a list of 29 symptoms from commonly reported symptoms (32) in addition to symptoms that were rarely reported in the literature but frequently mentioned by caregivers and patients in CP support groups.

### Caregiver Burden

The ZBI (Copyright 1980, 1983, 1990 Steven H Zarit and Judy M Zarit) is a 22-item self-report questionnaire completed by caregivers who were asked to rate their experiences on a 5-point Likert scale where 0 = never and 4 = nearly always. The 22-item ZBI encompasses questions related to caregiver health and psychological well-being, finances, impact on social life, and relationship with the individual with the disease. A global ZBI score was calculated for each participant consisting of the sum of all the caregiver ratings ranging from 0 (no burden) to 88 (highest burden). The range of scores from 0 to 20 are considered little or no burden, 21 to 40 are considered mild to moderate burden, 41 to 60 are considered high burden, and scores equal or above 61 considered severe burden (33). The term “relative” was used throughout ZBI in reference to the brain tumor survivor.

### Health-Related Quality of Life of Survivors

The PedsQL Pediatric Quality of Life Inventory (PedsQL, Lyon, France) is a modular instrument for measuring health-related quality of life (HRQOL) in persons with or without acute or chronic health conditions that encompasses 4 domain scores: physical, emotional, social, and school or work functioning. In the present study, we used the multidimensional 23-item caregiver-reported, age-dependent PedsQL 4.0 generic score scales assessment of HRQOL for young children (ages 5-7), children (ages 8-12), teens (ages 13-18), young adults (ages 18-25), and adults (ages 26 and above). Response choices were *never*, *almost never*, *sometimes*, *often*, and *almost always*. Items were reverse scored and linearly transformed to a scale from 0 (worst HRQOL) to 100 (best HRQOL). We used caregiver-reported HRQOL because the focus of the present study was to gather the caregiver perspective on survivor HRQOL.

### Statistical Analyses

Descriptive statistics are reported as N, percentages, mean (SD), and median. One-way analysis of variance (ANOVA) was conducted to compare the association of an individual’s age group or gender and caregiver burden or HRQOL. To examine relationships between variables, Pearson and Spearman correlation coefficients were used for normally and non-normally distributed outcomes, respectively. Group differences between survivors with and without a given individual health problem were assessed using independent Student *t* test or Mann-Whitney test for normally and non-normally distributed outcomes, respectively. For the Student *t* test, effect size is given by the Cohen *d*. For the Mann-Whitney test, effect size is given by the rank biserial correlation. The effect size was defined as trivial (<0.2), small (0.2-0.49), moderate (0.5-0.79), or large (≥0.8) (34). A Wilcoxon signed-rank test was used to compare PedsQL scores between functioning domains, because data was non-normally distributed. A linear regression model was used to examine the association between the number of health problems and ZBI or PedsQL scores and assess whether PedsQL scores predicted ZBI scores. All statistical analyses were computed using JASP Team (2022) (version 0.16.4, https://jasp-stats.org/download/).

## Results

### Sociodemographics of Participants

#### Caregivers

Caregivers (*N* = 82) were predominantly female (96%) with a mean (SD) of 48.3 (8.1) years (Table 1). Caregivers were predominately of European/White ethnic background (85%). Most were in a relationship (86%) and residing in the United States (77%). Respondents were highly educated with 65% of caregivers having a bachelor's or graduate degree. Most respondents were employed, either full-time (49%), part-time (21%), or self-employed (13%). The household annual gross income distribution was shifted toward higher income with 63% of caregivers reporting an income higher than $100 000. The mean (SD) duration of caregiving was 7.4 (6.0) years with a median of 5.5 years, a maximum of 28 years and a minimum of less than 1 year. It is worth noting that 82.8% of survivors aged ≥18 years lived with their family of origin, suggesting that caregiving extended into survivor’s adulthood.

**Table 1.** Sociodemographic characteristics of caregivers and CP survivors

Variable	Caregivers *n* = 82	Survivors *n* = 82	Variable	Survivors *n* = 82
Age in years, mean (SD)	48.3 (8.1)	16.7 (6.9)	Age category, *N* (%)	
Gender, *N* (%)			A young child age 5-7	4 (5)
Female	79 (96)	41 (50)	A child age 8-12	20 (24)
Male	3 (4)	41 (50)	A teenager age 13-17	29 (35)
Non-binary/transgender/other	0	0	A young adult age 18-25	21 (26)
Ethnic identity, *N* (%)			An adult age 26 years or older	8 (10)
African/Black	0	0	Primary living situation, *N* (%)	
Asian or Pacific Islander	4 (5)	3 (4)	Independent	2 (2)
European/White	70 (85)	61 (74)	With family of origin	75 (92)
Indigenous	0	0	With family of creation	0
Latin American or Hispanic	4 (5)	6 (7)	With partner/boyfriend/girlfriend	1 (1)
Other or mixed race	4 (5)	12 (15)	With multigenerational family	0
Marital status, *N* (%)			With nonrelative housemates	0
Single	7 (8)	81 (99)	Group home with support full-time	0
Partnered	3 (4)	1 (1)	Group home with support part-time	0
Married	67 (82)	0	Other	4 (5)
Separated or divorced	5 (6)	0	Able to financially support themselves, N (%)	
Widowed	0	0	Always	2 (2)
Country of residence, *N* (%)			Usually	3 (4)
USA	63 (77)	63 (77)	Sometimes	4 (5)
Canada	5 (6)	5 (6)	Rarely	5 (6)
UK	4 (5)	4 (5)	Never	19 (23)
Australia and New Zealand	6 (7)	6 (7)	N/A (survivor is a minor)	49 (60)
EU and non-UK Europe	3 (4)	3 (4)		
Asia and Pacific Islands	0	1 (1)		
Africa (S. Africa))	0	0		
Latin America and Caribbean Islands	0	0		
Middle East	1 (1)	0		
Other	0	0		
Highest level of education, *N* (%)				
Grade school	0	20 (24)		
Middle school	0	12 (15)		
Some high school	0	18 (22)		
High school	5 (6)	12 (15)		
Some college	24 (29)	13 (16)		
Bachelor's degree	26 (32)	5 (6)		
Graduate (master's or doctorate) degree	27 (33)	2 (2)		
Employment status, *N* (%)				
Full-time	40 (49)	2 (2)		
Part-time	17 (21)	15 (18)		
Self-employed	11 (13)	1 (1)		
Not currently employed	14 (17)	64 (78)		
Household annual gross income, *N* (%)				
Under $15 000	2 (2)			
Between $15 000 and $29 999	1 (1)			
Between $30 000 and $49 999	5 (6)			
Between $50 000 and $74 999	11 (13)			
Between $75 000 and $99 999	12 (15)			
Between $100 000 and $150 000	21 (26)			
Over $150 000	30 (37)			

The sociodemographic characteristics of the caregivers excluded from the analyses (*n* = 7) did not differ globally from those included in this analysis. There was, however, a higher representation of unemployed caregivers (*n* = 4) with a lower annual gross income (*n* = 5 reported an income below $100 000) (Supplementary Table S1 (35)).

#### Survivors

Most survivors were minors (64%) with a mean (SD) age of 16.7 (6.9) years (Table 1). The survivors’ ages were evenly distributed across childhood to early adulthood, except for young children aged 5 to 7 years and adults aged ≥26 years, who represented 5% and 10% of the population, respectively. Survivors were equally represented by gender with a 50% female/male ratio. Most survivors lived with family members (92%), including those aged 18 years and over (82.8%), whereas 3% lived independently or with a partner. Most survivors were European/Whites (74%). The highest level of education ranged from grade school (24%), middle school (15%), and some high school (22%).

### Clinical Characteristics of Survivors

Ninety-four percent (94%) of caregivers reported they were fully or partially responsible for survivor medical care while 6% reported that survivors had entire responsibility for their own care.

**Figure 1.**
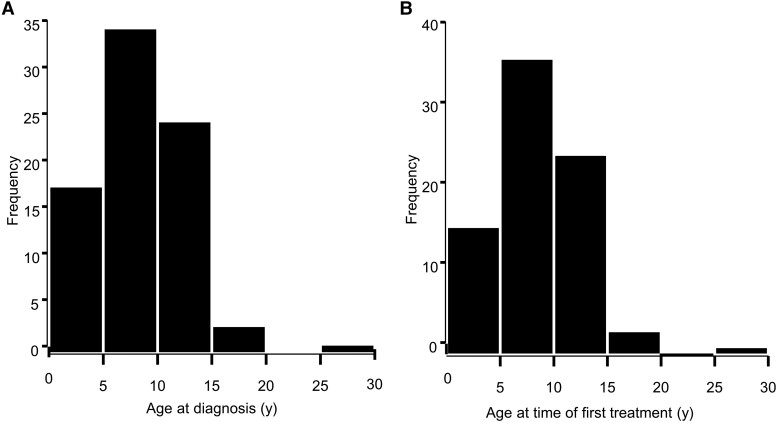
Frequency distribution of CP survivor age at (A) diagnosis and (B) age at time of first treatment. Age is expressed in years.

The distribution of survivor age at diagnosis and age at first treatment were skewed toward middle childhood (Fig. 1), with a mean (SD) of 9.3 (4.5), median of 9 years, and minimum-maximum range of 0 to 27 years and a mean (SD) of 9.3 (4.5), median of 9, and minimum-maximum range of 0 to 27 years, respectively. The time from diagnosis to first treatment was 1 year or less for 100% of survivors.

Fifty-five percent (55%) of survivors had no tumor recurrence (Supplementary Table S2A (35)), consistent with previous reports (11). Gross total resection (GTR) was the most common tumor treatment (52.4% of survivors), followed by partial resection surgery (PRS) with radiotherapy (32.9%) and proton therapy/radiation therapy (17.1%) (Supplementary Table S2B (35)). GTR was more frequent in survivors without tumor recurrence compared to those with recurrence (32.9% vs 18.3% survivors), while PRS with radiotherapy was more frequent in survivors with tumor recurrence compared to those without recurrence (22% vs 9.8% survivors) (Supplementary Fig. S1 (35)). These findings are consistent with prior studies (36, 37).

**Figure 2.**
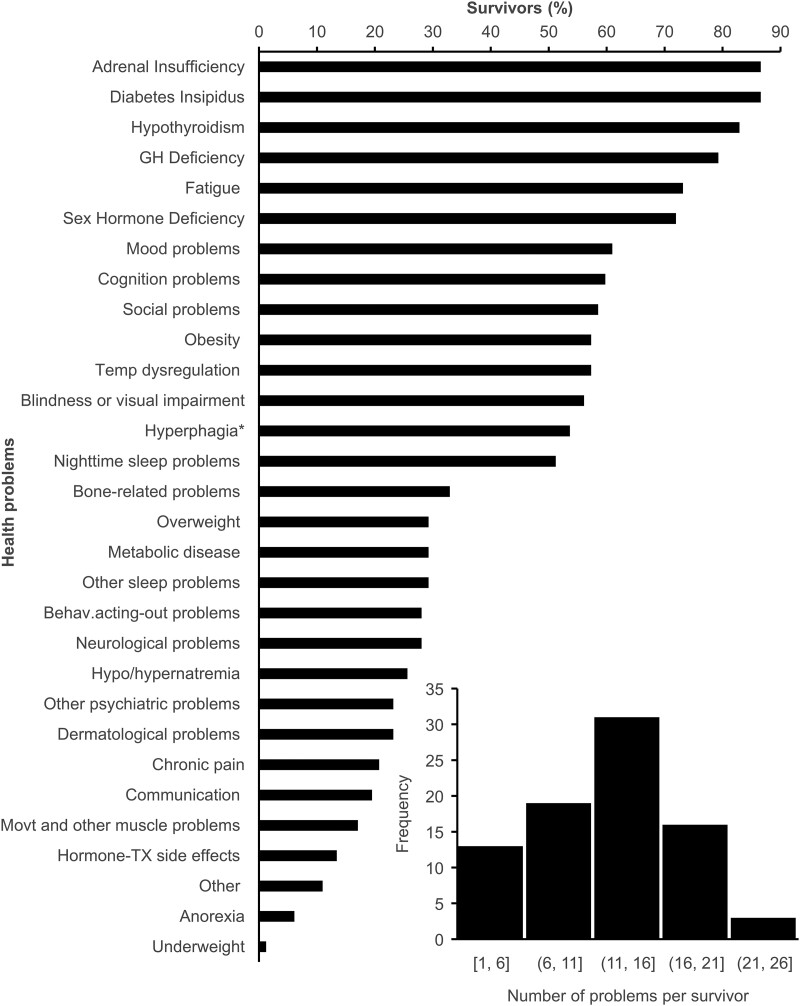
Health problems occurring after CP tumor treatment. Caregivers were asked to select all health problems that occurred following tumor treatments. Results are expressed as the % of survivors who selected a given problem. The lower right insert indicates the frequency distribution of the number of health problems that occurred per survivor. *Please note that excessive appetite/hunger, poor satiety, food seeking was indicated in the survey instead of hyperphagia. Similarly, lack of appetite/hunger, anorexia was indicated in place of anorexia. Obesity was defined as adults with BMI ≥ 30, children ≥ 95th percentile. Overweight was defined as adults with BMI = 25.0-29.9, children ≥85th to less than 95th percentile. Abbreviations: behav, behavioral; GH, growth hormone; movt: movement; temp, temperature; TX, treatment.

Caregivers were asked to select health problem(s) from a list of 29 symptoms to describe the medical problems that occurred in the survivors following tumor treatment(s). Caregivers reported a mean (SD) of 12.6 (5.7) health problems, with a minimum of 1 and a maximum of 27 problems (Fig. 2). The top (>70%) caregiver-reported problems experienced by survivors were weight problems: 87.8% (57.3% obese, 29.3% overweight, 1.2% underweight), adrenal insufficiency, diabetes insipidus, hypothyroidism, growth hormone deficiency, fatigue, and sex hormone deficiency (Fig. 2). Neurobehavioral problems including mood, cognition, and social problems were predominant (∼60%). Temperature dysregulation, blindness or visual impairment, and nighttime sleep problems affected over half of survivors. Hyperphagia affected 53.6% of survivors, whereas anorexia affected only 6.1%. Interestingly, 72% of survivors who experienced obesity also experienced hyperphagia, whereas 58.3% of survivors who experienced being overweight also had hyperphagia.

At the time of the survey, caregivers reported that survivors took an average (SD) of 6.88 (2.88) different medicines with a range of 1 to 13 different medicines per survivor. Hormone replacement therapies, including T4 thyroid hormone, corticosteroid, antidiuretic hormone, growth hormone, and sex hormone replacement, were the most frequent medication categories (Supplementary Fig. S2A (35)). Vitamin supplements were taken by half of survivors. Caregivers reported that only 19.5% of survivors took Food and Drug Administration (FDA)-approved weight loss medications (ie, phentermine and topiramate (Qsymia), liraglutide (Saxenda), semaglutide (Wegovy), orlistat (Xenical), bupropion-naltrexone (Contrave)). Physical therapy was the most common nonpharmacological treatment that survivors used since diagnosis (Supplementary Fig. S2B (35)). Outpatient psychotherapy, occupational therapy, and speech therapy were also quite frequent. Only 2 survivors had undergone bariatric surgery.

### Zarit Burden Interview

**Figure 3.**
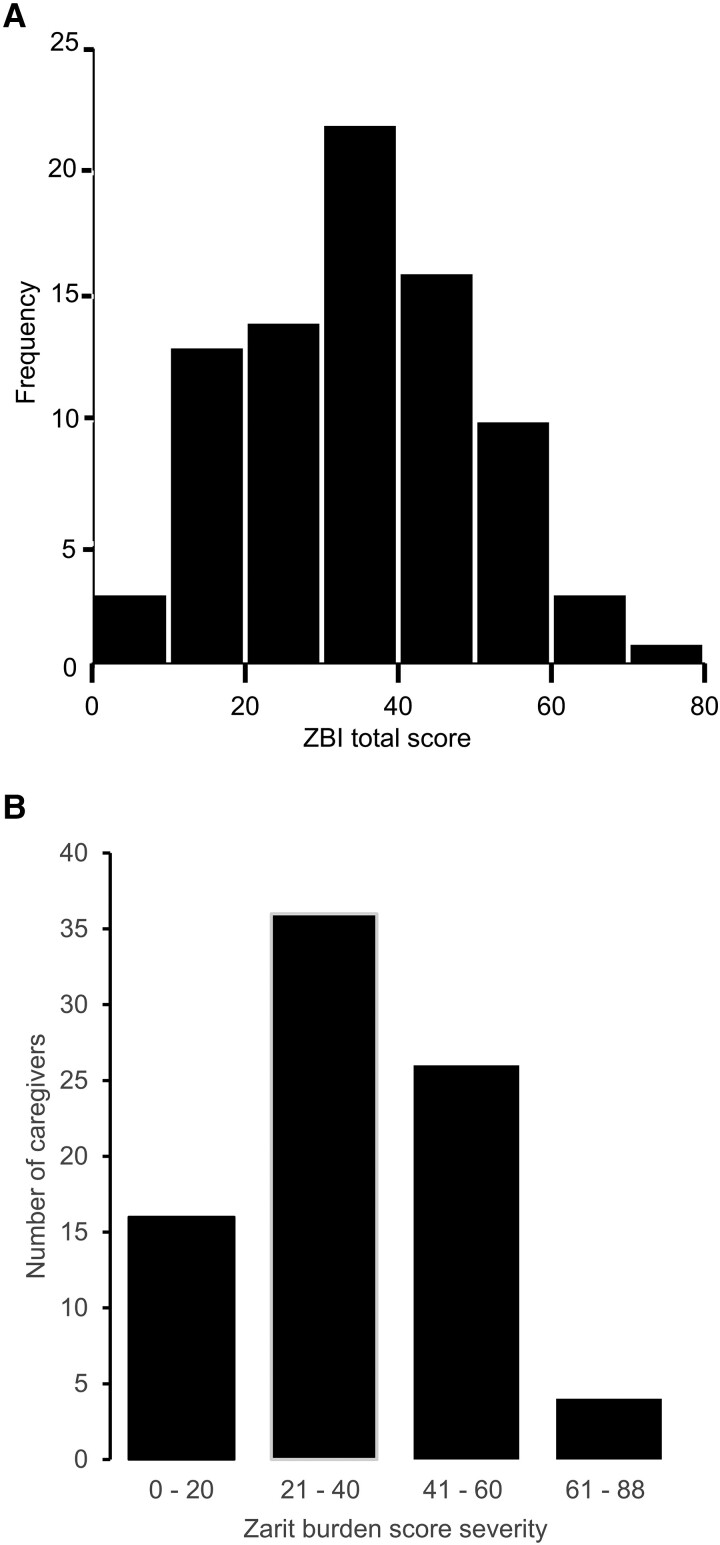
Zarit burden scores for caregivers of CP survivors. (A) Frequency distribution of ZBI total scores in CP caregivers. (B) Number of caregivers per category of burden severity: little or no burden (0-20), mild to moderate (21-40), moderate to severe (41-60), severe (61-88).

The ZBI scores had a bell-shaped distribution with scores ranging from 0 to 73 (Fig. 3A). The mean (SD) ZBI total score was 36.1 (15.6) and the median score of 37. Caregivers reported varying levels of burden, with 43.9% reporting mild to moderate burden, 31.7% moderate to severe burden, 4.9% severe burden, and only 19.5% reporting no or little burden (Fig. 3B). Notably, 75.6% of caregivers had ZBI scores above the cutoff score of 24, which has a significant predictive validity for identifying caregivers at risk for depression (38).

The levels of caregiver burden did not vary significantly across the 5 survivor age categories (F(4,77) = 0.738, *P* = .569), nor survivor gender (F(1,80) = 0.582, *P* = .448). In addition, ZBI scores were independent of survivor age, age at diagnosis, tumor recurrence, and care duration (Table 2). Although statistically significant, the relationship between caregiver burden and caregiver annual gross income was weak (Table 2).

**Table 2.** Relationship between caregiver burden and survivor's age, age categories, age at diagnosis, tumor recurrence, care duration, and caregiver’s household annual gross income

Variable		
Age	*r*	0.179#
	*P* value	.107
	95% CI	−0.04, 0.38
Age categories	*r*	0.155
	*P* value	.163
	95% CI	−0.06, 0.36
Age at diagnosis	rho	0.015#
	*P* value	.895
	95% CI	−0.20, 0.23
Tumor recurrence	*r*	−0.086#
	*P* value	.440
	95% CI	−0.30, 0.13
Care duration	*r*	0.193#
	*P* value	.083
	95% CI	−0.03, 0.39
Household annual gross income	*r*	−0.229#
	*P* value	**.038**
	95% CI	−0.43, −0.01

Correlation coefficients *r* represent Pearson’s coefficients except for Spearman’s *r* correlation indicated as #. *P* value < .05 is considered significant and indicated in bold.

**Figure 4.**
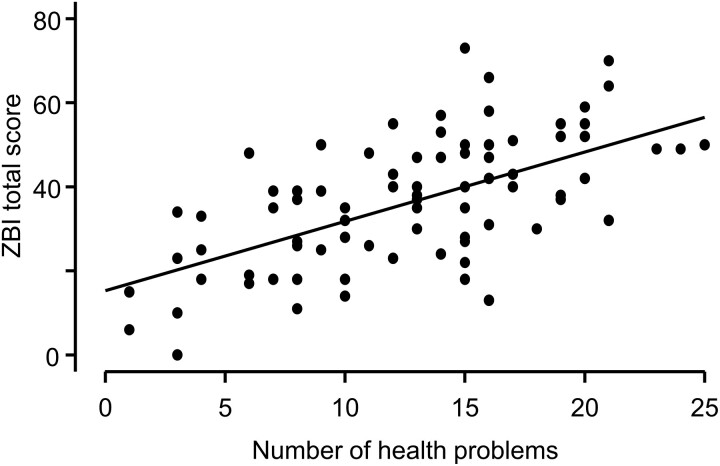
Scatter plot showing positive correlation between ZBI total score and number of health problems that occurred post tumor treatment.

To assess whether the level of caregiver burden was dependent on survivor health symptoms, we compared the mean ZBI scores between caregivers of survivors with and without a specific symptom occurring following tumor treatment(s) (Table 3). ZBI scores were statistically significantly higher in caregivers of survivors with symptoms indicated in Table 3. The effect sizes were large (≥0.8) for adrenal insufficiency, diabetes insipidus, temperature dysregulation, fatigue, sleep problems, behavioral acting out, social, and chronic pain. In contrast, ZBI scores were independent of growth hormone deficiency, dermatological problems, hormone-related treatment side effects, movement, and other muscle problems. Noteworthy, caregiver burden did not vary with survivor’s weight status or lack of appetite; rather, we found significantly higher level of burden measured in caregivers of survivors with hyperphagia. Further analysis showed a strong, positive, and significant correlation between ZBI scores and the number of symptoms each survivor experienced following tumor treatment(s) (*r*(80) = 0.61, *P* < .001) (Fig. 4). The number of health problems significantly predicted ZBI scores (F(1,80) = 46.75, *P* < .001), accounting for 36.9% of the variability in ZBI scores with adjusted *R*^2^ = 0.36.

**Table 3.** Zarit burden scores for caregivers of survivors with and without individual health symptoms listed

	Mean (SD), *N*				
Health symptoms	No	Yes	95% CI for mean difference	Effect size	*P* value
Adrenal insufficiency	20.8 (13.6), 11	38.5 (14.6), 71	−27.0, −8.0	−1.2	**<.001**
Diabetes insipidus	26.1 (18.7), 11	37.6 (14.6), 71	−23.0, −1.8	−0.8	**.021**
Hypo/Hypernatremia	35.0 (33.8), 61	43.0 (42.7), 21	−16.5, −1.2	−0.6	**.023**
Hypothyroidism	28.1 (14.9), 14	37.7 (15.3), 68	−18.5, −0.7	−0.6	**.035**
GH deficiency	32.6 (12.2), 17	37.0 (16.3), 65	−12.8, 4.1	−0.3	.309
Overweight	34.3 (15.6), 58	40.5 (15.1), 24	−13.7, 1.2	−0.4	.097
Anorexia	35.5 (15.1), 77	44.8 (22.1), 5	−23.5, 5.0	−0.6	.200
Temp dysregulation	28.3 (13.2), 35	41.9 (14.8), 47	−19.8, −7.2	−1.0	**<.001**
Fatigue	25.4 (13.8), 22	40.0 (14.4), 60	−21.7, −7.5	−1.0	**<.001**
Nighttime sleep problems	28.5 (14.9), 40	43.4 (12.6), 42	−21.0, −8.9	−1.1	**<.001**
Other sleep	31.8 (14.5), 58	46.4 (31.8), 24	−21.4, −7.7	−1.0	**<.001**
Blindness/visual impairment	31.9 (15.5), 36	39.4 (15.0), 46	−14.2, −07	−0.5	**.031**
Cognition	31.0 (15.1), 33	39.5 (15.1), 49	−15.3, −1.8	−0.6	**.014**
Behavioral acting out	31.3 (14.3), 59	48.4 (11.8), 23	−23.8, −10.4	−1.3	**<.001**
Mood	29.8 (16.5), 32	40.1 (13.7), 50	−17.0, −3.7	−0.7	**.003**
Social	28.9 (14.1), 34	41.2 (14.7), 48	−18.7, −5.8	−0.8	**<.001**
Communication problems	34.2 (15.5), 66	43.9 (13.7), 16	−18.1, −1.2	−0.6	**.025**
Other psychiatric problems	33.7 (14.3), 63	44.1 (17.4), 19	−18.3, −2.6	−0.7	**.010**
Neurological	33.6 (15.9), 59	42.4 (13.0), 23	−16.2, −1.4	−0.6	**.021**
Movt & other muscle problems	35.9 (15.5), 68	37.1 (16.4), 14	−10.4, 7.9	−0.1	.785
Bone-related problems	33.5 (15.5), 55	41.4 (14.6), 27	−15.0, −0.8	−.5	**.030**
Dermatological problems	34.6 (16.0), 63	41.0 (13.6), 19	−14.4, 1.7	−0.4	.119
Hormone-TX side effects	35.1 (16.1), 71	42.4 (10.1), 11	−17.2, 2.8	−0.5	.153
Chronic pain	33.1 (14.8), 65	47.6 (13.2), 17	−22.4, −6.6	−1.0	**<.001**
Obesity	32.5 (17.6), 35	38.8 (13.5), 47	−13.1, 0.4	−0.4	.068
Hyperphagia*^a^*	30.3 (16.7), 38	41.1 (12.8), 44	−17.3, −4.3	−0.7	**.001**
Sex hormone deficiency	28.7 (13.1), 23	39.0 (15.6), 59	−17.6, −2.9	−0.7	**.007**
Metabolic disease	33.9 (15.8), 58	41.4 (13.9), 24	−16.0, −2.0	−0.3	**.022#**

*P* values are derived from independent Student *t* test or Mann-Whitney test (#) for normally and non-normally distributed outcomes, respectively. For the Student *t* test, effect size is given by the Cohen *d*. For the Mann-Whitney test, effect size is given by the rank biserial correlation. *P* value < .05 is considered significant and indicated in bold.

Abbreviations: GH, growth hormone; Movt, movement; Temp, temperature; TX, treatment.

^*a*^Please note that excessive appetite/hunger, poor satiety, food seeking was indicated in the survey instead of hyperphagia.

### Health-Related Quality of Life

**Figure 5.**
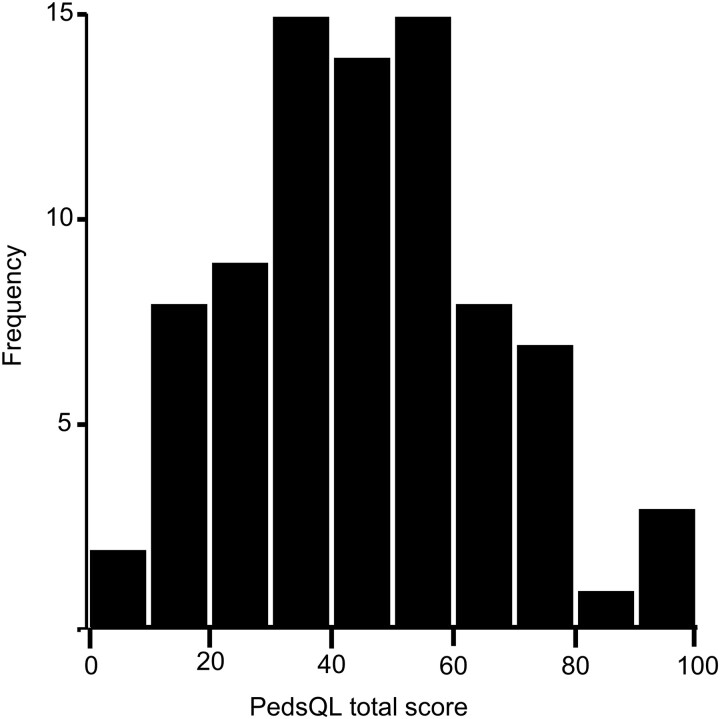
Frequency distribution of PedsQL total scores in CP survivors.

The overall mean (SD) PedsQL total score was 46.2 (20.9) with a median of 46.2. The low HRQOL scores are consistent with other studies (7, 12, 25, 39). The PedsQL total score had a bell-shaped distribution with scores ranging from 2.2 to 97.9 (Fig. 5). With respect to HRQOL dimensions, a Wilcoxon signed-rank test showed that the emotional functioning scores (median = 55) were significantly higher compared with the physical (median = 46.9, *z* = 1.99, *P* = .047), school/work (median = 45, *z* = 2.88, *P* = .004), and social functioning scores (median = 40, *z* = 3.61, *P* < .001). In contrast, there were no statistically significant differences between physical and school/work scores (*z* = 0.89, *P* = .372), physical and social scores (*z* = 1.26, *P* = .207), or school/work and social scores (*z* = 1.15, *P* = .250). Interestingly, we found strong correlations between all PedsQL functioning scores and total scores (Table 4), suggesting that all HRQOL domains are correlated and similarly impacted by CP tumors.

**Table 4.** Relationship between PedsQL total and functioning domains score, and ZBI total score

		PedsQL *n* = 82				
Variable		Physical functioning	Emotional functioning	Social functioning	School/Work functioning	ZBI total score *N* = 82
Physical functioning	*r*					−0.51
	*P* value					**<.001**
	95% CI					−0.65, −0.33
Emotional functioning	*r*	0.48				−0.57
	*P* value	**<.001**				**<.001**
	95% CI	0.29-0.63				−0.70, −0.40
Social functioning	*r*	0.69#	0.60			−0.67
	*P* value	**<.001**	**<.001**			**<.001**
	95% CI	0.56-0.79	0.44-0.72			−0.77, −0.53
School/Work functioning	*r*	0.79	0.56	0.80		−0.58
	*P* value	**<.001**	**<.001**	**<.001**		**<.001**
	95% CI	0.70-0.86	0.39-0.69	0.70-0.86		−0.71, −0.42
PedsQL total score	*r*	0.91	0.72	0.89	0.91	−0.66
	*P* value	**<.001**	**<.001**	**<.001**	**<.001**	**<.001**
	95% CI	0.86-0.94	0.60-0.81	0.84-0.94	0.87-0.94	−0.77, −0.51

Correlation coefficients *r* represent Pearson coefficients except for Spearman *r* correlation indicated as #. *P* value < .05 is considered significant and indicated in bold.

We examined determinants of survivor HRQOL and found that only the number and type of survivor health issues were factors affecting survivor HRQOL (Supplementary Table S3, Supplementary Fig. S3 (35)). In addition to dermatological problems, the same health problems associated with high ZBI scores were associated with low PedsQL scores (Supplementary Table S4 (35)).

### Relationship Between Survivor Health-Related Quality of Life and Caregiver Burden

**Figure 6.**
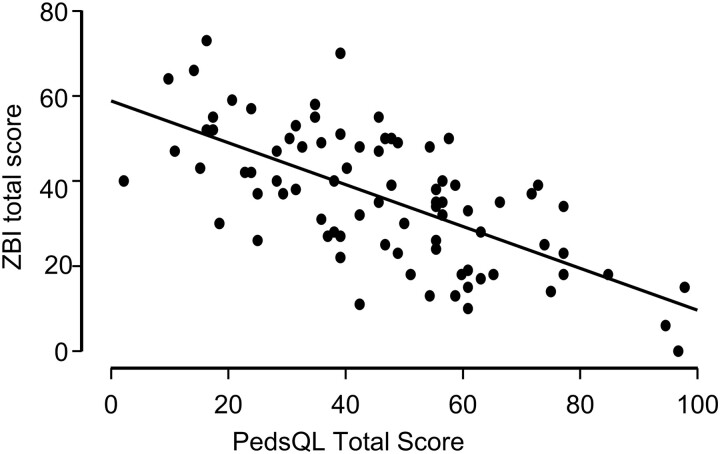
Relationship between HRQOL and caregiver burden. Shows survivor PedsQL total score in function of ZBI total score for each of the 82 caregivers of the study.

We found a strong, inverse, and significant correlation between PedsQL and ZBI total scores (*r*(80) = −0.66, *P* < .001) (Fig. 6). Likewise, all PedsQL functioning domains and ZBI total scores were strongly, inversely, and significantly correlated (Table 4). PedsQL total scores significantly predicted ZBI scores (F(1,80) = 60.90, *P* < .001, adjusted *R*^2^ = 0.43) with the regression equation: *y* = 58.800 − 0.492*x* (HRQOL). For each unit increase in PedsQL total score, ZBI total score decreased by about 0.62 to 0.37 points (B = −0.49; 95% CI: −0.62, −0.37).

## Discussion

This study measured the subjective burden for caregivers of CP survivors and explored determinants of burden in relation to caregiver-reported survivors’ clinical manifestations and HRQOL. Overall, we found that caregivers reported moderate levels of caregiver burden and poor survivor HRQOL scores. Caregiver burden was independent of caregiver income, survivor age, age at diagnosis, care duration, gender, and tumor recurrence. In contrast, ZBI scores were associated with the number and type of health problems affecting survivors following tumor treatment. Interestingly, we found that caregiver burden and survivor HRQOL were strongly and inversely correlated, and that survivor HRQOL predicted the level of caregiver burden of CP survivors. To our knowledge, this is the first study to measure caregiver experience of burden in CP and to give insight into the factors contributing to caregiver burden. These data shed light on critical unmet needs for CP caregivers and survivors and open avenues for the development of new treatments.

Most caregivers in our study were middle-aged, White, female, from a higher socioeconomic background, and residing in the United States. Survivors were primarily teenagers with childhood-onset CP. Notably, more than 80% of survivors aged ≥18 resided with family and nearly all survivors were medically dependent on their caregivers. These findings highlight the long-term and specialized care needed for survivors, extending into adulthood, and the crucial role of their caregivers in providing this care.

Following tumor treatment, caregivers reported an average of 13 out of 29 health conditions in survivors, with high rates of fatigue, various hypopituitarism disorders, mood, cognition, social issues, temperature dysregulation, visual impairment, and nighttime sleep problems underlying the CP poly-symptomatology in concordance with previous studies (12, 32, 40, 41). We found that overweight and obesity affected most survivors, consistent with previous research (23, 24, 32). Strikingly, our study revealed that 70% of survivors who experienced obesity also experienced hyperphagia. To the best of our knowledge, this is the first study to measure the proportion of CP survivors with hyperphagia.

Hyperphagia is a multifaceted concept that has been well characterized in monogenic and polygenic obesity (42) and defined by clinical features that manifest in patients as unrelenting appetite, impaired satiety, intense preoccupation with food and excessive food-seeking behavior including nocturnal eating, food stealing and stashing, and stress and anxiety when food is denied (43). Although hyperphagia is commonly reported (39, 44, 45), there has been little attempt to characterize hyperphagia in CP survivors beyond an uncontrolled appetite (43) and abnormal food-seeking behavior (46). The lack of validated measures capturing the multidimensional aspects of hyperphagia (42) poses additional challenges. In this context, we believe that the caregiver report of survivor excessive appetite and/or hunger, poor satiety, and food seeking was the best method to assess hyperphagia in CP. Future research is needed to characterize and validate methods to assess hyperphagia in CP.

Given the high prevalence of panhypopituitarism in CP survivors, it was not surprising to find that hormone replacement therapies were the most utilized medications, as previously described (13). In contrast, whereas approximately 60% of survivors in the current study were impacted by neurobehavioral problems, effective treatments for these issues are lacking (7, 25). Interestingly, despite the high prevalence of obesity, fewer than 20% of survivors were taking FDA-approved weight loss medications and only 2 survivors had received bariatric surgery. While it is possible that off-label use of weight loss medications for HO is limited due to lack of insurance reimbursement (47) or that they lead to suboptimal results not reported in the literature, HO remains difficult to treat and is a critical unmet need (48, 49).

The levels of caregiver burden for CP survivors, as measured by the mean ZBI scores, were found to be higher than those observed in caregivers for persons with dementia (50), Alzheimer disease (51), and chronic pain (52). Interestingly, the levels of caregiver burden in CP and acquired brain injury were similar (53). Several factors may contribute to the higher caregiver burden for CP survivors and people with acquired brain injury, including the difficulty of caring for people with a lifelong condition, the nature of the relationship between the caregiver and the patient (ie, child vs adult), and the type and severity of symptoms affecting the patient (53). ZBI scores were, however, lower than those measured in Prader-Willi syndrome (54). This result is surprising given the many overlapping traits shared by these 2 hypothalamic syndromes (20). This difference in scores could reflect the disparity between caring for a person with a genetic neurodevelopmental disorder characterized by severe clinical manifestations from birth, as opposed to caring for a person with a condition often diagnosed later in childhood (9.3 years in this study) and whose clinical severity and manifestations vary significantly with tumor size, tumor location, and treatments (11). Nonetheless, based on the ZBI cutoff score defined by Schreiner, about 80% of caregivers in our study could be at risk for depression (38), warranting further assessment of QOL for caregivers of CP survivors (55).

In contrast to previous studies, we found no significant association between caregiver burden and caregiver level of education, number of years providing care, income, survivor age, age at diagnosis, or tumor recurrence (56, 57). Instead, we found that caregiver burden was associated with the number and type of health conditions affecting the survivor following tumor treatment(s). Notably, symptoms of hypothalamic dysfunction, such as hypo- or hypernatremia accompanying diabetes insipidus, temperature dysregulation, sleep problems, hyperphagia, and impaired behavioral and social functioning, were the most frequent health issues linked to high caregiver burden. These results extend the previously reported negative impact of hypothalamic dysfunction on CP survivors’ QOL to the level of burden experienced by their caregivers. Strikingly, survivor hyperphagia significantly impacted caregiver burden reaching levels seen in caregivers of people with Prader-Willi syndrome (54). In contrast, survivor obesity did not significantly impact caregiver burden. This suggests that, like in Prader-Willi syndrome (58), hyperphagia in CP survivors is a stronger determinant of caregiver burden than obesity. Other issues, although less frequent, were also associated with high caregiver burden, including communication impairment, neurological and psychiatric conditions, chronic pain, bone-related issues, and metabolic diseases. Further research should be undertaken to examine whether the high caregiver burden associated with these issues could result from the lack of effective treatments.

The present study provides 3 levels of evidence to support caregiver-reported survivor HRQOL as a critical determinant of caregiver burden in CP. First, we showed that the number and type of health issues were found to be critical determinants for survivor HRQOL and for caregiver burden. Second, we found that all health problems associated with low HRQOL except for dermatological issues were also associated with increased caregiver burden. Noteworthy, contrary to previous findings (59), our study found that hyperphagia, not obesity, significantly impacted HRQOL. Third, we found a strong and inverse correlation between caregiver burden and survivor HRQOL and showed that HRQOL is a good predictor of caregiver burden.

The correlation between survivor PedsQL and ZBI scores suggests that addressing CP symptoms may improve the QOL of survivors and reduce caregiver burden. This finding highlights the potential use of the ZBI as an exploratory outcome measure in clinical trials to evaluate the effect of interventions on both CP survivors and their caregivers. In addition to improving the well-being of caregivers, reducing caregiver burden could significantly decrease the economic costs incurred for CP survivors. A comprehensive cost evaluation of CP from a societal perspective is necessary, considering the patient journey from diagnosis to treatment, interventions, informal care, and QOL (60).

Our study has several limitations. First, it is cross-sectional and does not capture the trajectory of caregiver burden and survivor HRQOL over time, making causal conclusions challenging. Second, our sample's sociodemographics are not representative of adult CP survivors or broad ethnic groups. Therefore, our findings may not generalize to these populations and future research should address these limitations. Third, we cannot exclude a self-selection bias toward caregivers of survivors with symptoms on the most severe spectrum. Fourth, our study did not differentiate between direct and indirect effects of health issues on HRQOL or caregiver burden (25). Fifth, tumor location has a known impact on survivor QOL (15, 61), but our inability to access medical records due to the anonymity of the survey prohibited verification of this information. In addition, we cannot exclude errors in clinical data reported by caregivers since they were not corroborated by medical records.

Altogether, our results suggest that caregiver burden in CP is significant and associated with the survivor's symptomatology impacting HRQOL, not the caregiver's characteristics or caregiving duration. Our study showed that caregiver assessment of survivor HRQOL is a valuable tool to measure the overall impact faced by caregivers of CP survivors. The study findings also stress the significance of the poly-symptomatology induced by the tumor and/or treatments and the cumulative effect of the symptoms on both survivors and their caregivers. Of particular importance, our study separated hyperphagia and obesity and identified hyperphagia as a critical unmet need in a large subset of survivors. More generally, this result questions the restrictive focus of new therapeutic developments on HO rather than on symptoms of hypothalamic dysfunction including temperature dysregulation, behavioral acting out, sleep problems, social impairment, fatigue, and psychiatric problems. Our study also highlights the importance of collecting both patient and caregiver related impact of CP to better identify critical determinants of health, HRQOL, and caregiver burden. Future interventions should focus on caregivers in addition to survivors, with particular attention to caregiver perceptions of the long-term sequelae for CP survivors.

## Data Availability

Some or all datasets generated during and/or analyzed during the current study are not publicly available but are available from the corresponding author on reasonable request.

## Abbreviations

CP: craniopharyngioma
GTR: gross total resection
HO: hypothalamic obesity
HRQOL: health-related quality of life
PedsQL: Pediatric Quality of Life Inventory
PRS: partial resection surgery
QOL: quality of life
ZBI: Zarit Burden Interview
